# Factors influencing mothers’ health care seeking behaviour for their children: evidence from 31 countries in sub-Saharan Africa

**DOI:** 10.1186/s12913-020-05683-8

**Published:** 2020-09-07

**Authors:** Sulaimon T. Adedokun, Sanni Yaya

**Affiliations:** 1 Department of Demography and Social Statistics, Obafemi Awolowo University, Ile-Ife, Nigeria; 2 School of International Development and Global Studies, Faculty of Social Sciences, University of Ottawa, 120 University Private, Ottawa, ON K1N 6N5 Canada; 3 The George Institute for Global Health, The University of Oxford, Oxford, UK

**Keywords:** Health care, Children, Diarrheal, Seeking behaviour, Mother, Global Health, Demographic and health surveys

## Abstract

**Background:**

Almost half of the estimated 5.3 million deaths of under-five children in 2018 occurred in sub-Saharan Africa with morbidity contributing substantially to these deaths. Seeking medical care for children has been described as an important measure of reducing mortality occasioned by morbidity. This study examined factors influencing mothers’ health seeking behaviour for their children in sub-Saharan Africa.

**Methods:**

This study made use of data from Demographic and Health Surveys (DHS) of 31 countries in sub-Saharan Africa. The study involved 75,982 children who received or did not receive measles vaccine and 93,142 children who sought or did not seek medical care when affected by fever or cough and diarrhoea. Binary logistic regression was applied in the analysis.

**Results:**

Most of the children (74%) received measles vaccine while less than one-fifth sought medical care for fever or cough (16%) and diarrhoea (10%). Majority of the children of women who received measles vaccine and sought medical care when they had fever or cough are from richest households. Children of women with primary and secondary or higher education, children of working women and children of women that attended antenatal care during pregnancy are more likely to seek medical care for fever or cough. While children of women who live in urban areas and children of second or higher order of birth are less likely to receive measles vaccine, children aged 24–35 months and those who were of average size at birth are less likely to seek medical care for diarrhoea.

**Conclusions:**

This study has revealed that mothers’ health care seeking behaviour for their children is influenced by social, maternal and child factors. Any intervention aimed at improving child health in sub-Sharan Africa should take these factors into consideration.

## Background

Although global progress has been made in reducing child mortality, an estimated 5.3 million under-five children died in 2018 with almost half of these deaths occurring in sub-Saharan Africa [1]. Reports indicate that children in sub-Saharan Africa are more than 15 times more likely to die before age 5 than their counterparts in high income countries [2]. Morbidity contributes substantially to these deaths as pneumonia, malaria and diarrhoea have been linked to about 29% of global deaths of under-five children in 2018 [3]. While malaria resulted in approximately 266,000 deaths of these children, diarrhoea is responsible for 480,000 deaths of young children across the world [3]. In 2018, about 140,000 measles deaths occurred globally, mostly among under-five children [4].

Seeking medical care for sick children is an important aspect of child health. Many programmes have been established in order to ensure that children receive adequate care during illness. One of such programmes is Integrated Management of Childhood Illness (IMCI) which aims at providing direction to the world child health community on the best ways to assist countries in ensuring child survival [5]. The programme has three components: (i) improving case management skills of health care staff (ii) improving overall health systems (iii) improving family and community health practices. By extension, materials were produced to assist community health workers assess and treat sick children who are between 2 and 59 months [6]. In the same vein, UNICEF introduced the Strategy for Health 2016–2030 with two overarching goals: putting an end to preventable maternal, newborn and child deaths and promoting the health and development of all children [7]. In order to achieve these goals, three key approaches were highlighted. These approaches include tackling inequities in health outcomes, strengthening health system, including emergency preparedness, response and resilience and promoting integrated, multi-sectoral policies and programmes [7].

However, despite these efforts at promoting child health, many mothers or caregivers do not seek medical care for their children [8]. This invariably constitutes threat to the survival of such children. Many factors have been linked to poor health care utilization for children. These factors include education, child’s age, residence, perceived importance of early treatment, mass media exposure, level of income, birth order, socioeconomic status, occupational status [8–15]. Most of these studies focused attention on factors influencing health seeking behaviour for children in individual countries. There is a need for studies that would consider factors at regional or sub-regional level. This study aimed at filling this gap by examining mothers’ health seeking behaviour for children and associated factors in sub-Saharan Africa as a whole.

## Methods

### Study design

This cross-sectional study was conducted in accordance with the STROBE (STrengthening the Reporting of OBservational studies in Epidemiology) recommendations. The data used in this study were obtained from Demographic and Health Surveys (DHS) of 31 countries in sub-Saharan Africa. The survey provides information on health and population issues. The countries were selected based on their most recent surveys. In order to ensure that the analysis is robust, we limited the data sets to surveys conducted between 2010 and 2018. Table 1 shows the countries, years of survey, number of children and sex of children involved in the study.

**Table 1:** Year of survey, number of children and percentage of child sex by country in Sub-Saharan Africa

Country	Year of survey	Number of children	Child sex in percentage	
			Male	Female
Angola	2015–2016	3316	50.6	49.4
Benin	2017–2018	6042	50.0	50.0
Burkina Faso	2010	3357	51.9	48.1
Burundi	2016–2017	3037	50.0	50.0
Cameroon	2011	2744	49.0	51.0
Chad	2014–2015	4892	49.5	50.5
Comoros	2012	1.416	50.2	49.8
Congo	2011–2012	2345	51.2	48.8
Cote d’Ivoire	2011–2012	1795	50.2	49.8
Democratic Republic of Congo	2013–2014	4195	50.8	49.2
Ethiopia	2016	4575	50.7	49.3
Gabon	2012	1897	49.9	50.1
Gambia	2013	1818	51.8	48.2
Ghana	2014	1395	52.3	47.7
Guinea	2018	1609	52.6	47.4
Kenya	2014	9526	51.1	48.9
Lesotho	2014	721	49.2	50.8
Liberia	2013	1698	53.3	46.7
Malawi	2015–2016	2625	48.4	51.6
Mali	2018	4320	49.9	50.1
Namibia	2013	1009	48.6	51.4
Niger	2012	2658	50.7	49.3
Nigeria	2018	5713	52.0	48.0
Rwanda	2014–2015	1858	51.6	48.4
Senegal	2010–2011	2159	54.0	46.0
South Africa	2016	691	51.8	48.2
Tanzania	2015–2016	4715	50.4	49.6
Togo	2013–2014	1667	49.2	50.8
Uganda	2016	2270	51.5	48.5
Zambia	2018	4509	49.7	50.3
Zimbabwe	2015	2570	49.7	50.3

### Sampling technique

Sample selection in the surveys involved multi-stage cluster sampling method. Primary sampling unit (PSU) was adopted which divided each country into communities or clusters. Such clusters were further carved out from urban and rural areas. In each urban and rural area, households were selected for the survey. The survey involved women aged 15 to 49 years who are either permanent residents of households or visitors present at the time of the interview.

### Data collection

Standardized questionnaires were used to obtain information from women aged 15–49 years. The method of data collection involved face-to-face interview with the respondents. To ensure that information was well collected, each interviewer administered a set of questionnaires in which every information provided by the respondents was recorded. Information about the children was obtained from the mothers. Some of the questions focused on socioeconomic characteristics, reproductive history, antenatal and delivery care, child health, among others.

### Outcome variable

Three outcome variables have been used in this study. The first outcome variable involved children aged 12–23 months who received or did not receive measles vaccine. Those who received measles vaccine were categorized as ‘Yes’ and those who did not receive the vaccine were categorized as ‘No’. The second outcome variable is whether medical care was sought when the children had fever or cough. Children in this category were between ages 7 and 35 months. Those who sought medical care were categorized as ‘Yes’ and while those who did not seek medical care were categorized as ‘No’. The third outcome variable is whether medical care was sought when children had diarrhoea. This also involved children aged 7–35 months. Those who sought medical care were categorized as ‘Yes’ and those who did not seek medical care were categorized as ‘No’.

### Independent variables

The independent variables included in the study are mother’s age, mother’s education, household wealth, employment status, residence, child sex, child’s age, birth order, size of child at birth and antenatal care attendance. Mother’s age is categorized into 15–24 years, 25–34 years and 35 years and above. Education was grouped into none, primary and secondary or higher. Household wealth was divided into five quintiles: poorest, poorer, middle, richer and richest. Employment status was defined as working and not working. Residence was categorized as rural and urban. Child sex was measured as male and female. Child’s age has three categories, namely; 7–11 months, 12–23 months and 24–35 months. Birth order was grouped into first order birth, second order birth and third order birth and above. Size of child at birth was defined as large, average and small. Antenatal care has two categories: did not attend and attended.

### Statistical analysis

Analysis of the data in this study involved three stages. In the first stage, weighting factor was applied to the data set of each country before pooling them tighter to arrive at a single data set for sub-Sharan Africa. This was done in order to address the problem of under-enumeration and over-enumeration in the surveys. The second stage is the descriptive statistics expressed in number and percentages. At this stage, Chi-Square test was used to determine the relationships between independent variables and the outcome variables. To make the analysis easy and results obtained therefrom easily interpretable, data for the 31 countries were pooled together. The third stage involved application of binary logistic regression. It is a statistical technique used when the outcome variable is dichotomous while the independent variables may be of any form. In this case, each outcome variable is dichotomized. For instance, children who received measles vaccine were coded 1 while those who did not receive the vaccine were coded 0. Children who sought medical care when they had fever or cough were coded 1 and those who did not seek medical care were coded 0. Children who sought medical care when they had diarrhoea were coded 1 and those who did not seek medical care were coded 0. The results were interpreted using odds ratio, 95% confidence intervals and a significance level of 0.05.

## Results

### Descriptive statistics

Table 1 captures demographic characteristics of respondents. The study involved 75,982 children who received or did not receive measles vaccine and 93,142 children who sought or did not seek medical care when affected by fever or cough and diarrhoea. Most of the children (74%) received measles vaccine while less than one-fifth of the children sought medical care for fever or cough (16%) and diarrhoea (10%). With respect to the independent variables (Table 2), most of the children whose mothers had secondary or higher education received measles vaccine (86%). However, majority of the children whose mothers had primary education sought medical care when they had fever or cough and diarrhoea (19 and 11% respectively). Majority of the male and female children received measles vaccine. There is no significant difference in the percentage of male and female children that sought medical care when they had fever or cough and diarrhoea. Most of the children who received measles vaccine and sought medical care when they had fever or cough are from the richest households (85 and 17% respectively). As for medical care in respect of diarrhoea, most of the children are from poorer household (11%). Majority of the children that received measles vaccine and sought medical care when they had fever or cough live in urban areas. Most of the children that sought medical when they had fever or cough and diarrhoea were between 7 and 11 months (18 and 13% respectively). Most of the children that received measles vaccine, sought medical care when they had fever or cough and diarrhoea belong to the first order birth category. The mothers of the majority of children who received measles vaccine and sought medical care for fever or cough and diarrhoea attended antenatal care.

**Table 2:** Relationship between covariates and child health in Sub-Saharan Africa

Covariates	Received measles vaccine (*n* = 75,982)		Sought medical treatment for fever/cough (*n* = 93,142)		Sought medical treatment for diarrhoea (*n* = 93,142)	
Mother’s age	No	Yes	No	Yes	No	Yes
15–24	26.9	73.1	83.0	17.0	88.7	11.3
25–34	25.1	74.9	84.1	15.9	90.3	9.7
35+	25.4	74.6	85.3	14.7	91.5	8.5
Education						
None	36.6	63.4	88.1	11.9	91.4	8.6
Primary	21.7	78.3	80.9	19.1	88.6	11.4
Sec/higher	14.4	85.6	81.9	18.1	89.8	10.2
Household wealth						
Poorest	34.4	65.6	84.6	15.4	89.8	10.2
Poorer	27.7	72.3	83.9	16.1	89.4	10.6
Middle	24.3	75.7	83.8	16.2	90.1	9.9
Richer	21.3	78.7	84.1	15.9	90.1	9.9
Richest	14.9	85.1	83.1	16.9	91.0	9.0
Employment						
Not working	27.3	72.7	85.1	14.9	89.8	10.2
Working	24.6	75.4	83.2	16.8	90.2	9.8
Residence						
Rural	27.9	72.1	84.1	15.9	89.8	10.2
Urban	20.7	79.3	83.7	16.3	90.4	9.6
Child sex						
Male	25.6	74.4	83.8	16.2	89.6	10.4
Female	25.8	74.2	84.2	15.8	90.4	9.6
Child’s age						
7–11 months			82.0	18.0	87.4	12.6
12–23 months	27.2	72.8	83.1	16.9	88.4	11.7
24–35 months	24.1	75.9	85.9	14.1	93.0	7.0
Birth order						
1st order	23.6	76.4	83.3	16.7	89.8	10.2
2nd order	27.5	72.5	84.8	15.2	90.0	10.0
3rd order or more	30.8	69.2	85.3	14.7	90.9	9.1
Size of child at birth						
Large	26.4	73.6	83.5	16.5	89.6	10.4
Average	24.4	75.6	85.2	14.8	90.9	9.1
Small	30.4	69.6	84.0	16.0	88.7	11.3
ANC attendance						
Did not attend	41.3	58.7	89.9	10.1	94.1	5.9
Attended	20.1	79.9	82.1	17.9	88.7	11.3

### Multivariate analysis

Results from the multivariate analysis (Tables 3, 4 and 5) show that children of whose mothers were 25 years and above and those who had primary and secondary or higher education are more likely to receive measles vaccine. Household wealth also shows that children from poorer, middle, richer and richest households are most likely to receive measles vaccine. Children who live in urban areas and children of second or higher birth order are less likely to receive measles vaccine. Children whose mothers are working and those who attended antenatal care are more likely to receive measles vaccine. Children who are of average size at birth are 14% more likely to receive measles vaccine compared to children who are large at birth.

**Table 3:** Logistic regression of correlates of measles vaccination (12–23 months) for children in Sub-Saharan Africa

Variable	Received measles vaccine	
	Odds ratio	95% Confidence interval
Sex of child		
Male	0.99	0.96–1.04
Female	1	
Mother’s age		
15–24	1	
25–34	1.22*	1.16–1.28
35+	1.42*	1.32–1.52
Education		
None	1	
Primary	1.77*	1.69–1.84
Sec/higher	2.52*	2.39–2.66
Household wealth		
Poorest	1	
Poorer	1.21*	1.15–1.27
Middle	1.37*	1.29–1.44
Richer	1.52*	1.44–1.61
Richest	2.09*	1.95–2.26
Employment		
Not working	1	
Working	1.09*	1.06–1.14
Residence		
Rural	1	
Urban	0.83*	0.79–0.87
Child’s age		
7–11 months		
12–23 months	1	
24–35 months	1.48*	1.43–1.54
Birth order		
1st order	1	
2nd order	0.88*	0.84–0.93
3rd order or more	0.79*	0.73–0.84
Size of child at birth		
Large	1	
Average	1.14*	1.09–1.19
Small	0.96	0.92–1.02
ANC attendance		
Did not attend	1	
Attended	2.60*	2.50–2.70

*Level of significance at *p*<0.05

**Table 4:** Logistic regression of correlates of medical treatment for fever/cough (7–35 months) among children in Sub-Saharan Africa

Variable	Sought medical treatment for cough/fever	
	Odds ratio	95% Confidence interval
Sex of child		
Male	1.04	0.99–1.08
Female	1	
Mother’s age		
15–24	1	
25–34	0.96	0.92–1.01
35+	0.88*	0.82–0.95
Education		
None	1	
Primary	1.54*	1.48–1.62
Sec/higher	1.49*	1.42–1.57
Household wealth		
Poorest	1	
Poorer	0.98	0.92–1.03
Middle	0.98	0.93–1.04
Richer	0.95	0.89–1.01
Richest	0.99	0.93–1.07
Employment		
Not working	1	
Working	1.25*	1.20–1.29
Residence		
Rural	1	
Urban	0.92*	0.88–0.96
Child’s age		
7–11 months	1	
12–23 months	0.94*	0.89–0.99
24–35 months	0.84*	0.79–0.89
Birth order		
1st order	1	
2nd order	0.99	0.94–1.04
3rd order or more	1.04	0.97–1.13
Size of child at birth		
Large	1	
Average	0.88*	0.85–0.92
Small	1.04	0.98–1.09
ANC attendance		
Did not attend	1	
Attended	1.68*	1.59–1.77

*Level of significance at *p*<0.05

**Table 5:** Logistic regression of correlates of medical treatment for diarrhoea (7–35 months) among children in Sub-Saharan Africa

Variable	Sought medical treatment for diarrhoea	
	Odds ratio	95% Confidence interval
Sex of child		
Male	1.11*	1.06–1.16
Female	1	
Mother’s age		
15–24	1	
25–34	0.86*	0.81–0.91
35+	0.72*	0.66–0.79
Education		
None	1	
Primary	1.27*	1.20–1.34
Sec/higher	1.19*	1.12–1.27
Household wealth		
Poorest	1	
Poorer	0.99	0.93–1.06
Middle	0.94	0.88–1.01
Richer	0.93	0.87–1.004
Richest	0.83*	0.76–0.91
Employment		
Not working	1	
Working	0.96	0.92–1.005
Residence		
Rural	1	
Urban	0.96	0.90–1.02
Child’s age		
7–11 months	1	
12–23 months	0.95	0.90–1.01
24–35 months	0.61*	0.57–0.65
Birth order		
1st order	1	
2nd order	1.14*	1.04–1.26
3rd order or more	1.15*	1.04–1.26
Size of child at birth		
Large	1	
Average	0.86*	0.82–0.90
Small	1.13*	1.06–1.21
ANC attendance		
Did not attend	1	
Attended	1.79*	1.68–1.92

*Level of significance at *p*<0.05

For fever or cough, results show that children whose mothers had primary and secondary or higher education, are working and attended antenatal care are more likely to seek medical care for cough or fever. However, children whose mothers are 35 years and above and those living in urban areas are less likely to seek medical care for fever or cough. Also, children who are 12–23 months and above and those who are of average size at birth are less likely to seek medical care.

With respect to diarrhoea, children who are small at birth, whose mothers had primary and secondary or higher education and attended antenatal care are more likely to seek medical care for diarrhoea. Also, children of second and third order of birth or more are 14 and 15% respectively more likely to seek medical care for diarrhoea. Male children are 11% more likely to seek medical care compared to female children. Children whose mothers are 25 years and above and those from the richest households are less likely to seek medical care for diarrhoea. At the same time, children who are 24–35 months and those who are of average size at birth are less likely to seek medical care for diarrhoea.

## Discussion

This study has revealed the factors which influence mothers’ health care seeking behaviour for their children in sub-Saharan Africa. Results from the study show that mothers’ age has a significant effect on mothers’ health care seeking behaviour for their children. Children **whose mothers** are 25 years and above are more likely to receive measles vaccine compared to children of women who are 15–24 years. Results further show that this category of women (25 years and older) are less likely to seek medical care for their children whenever they are down with fever or cough and diarrhoea [16–18]. This indicates that younger women tend to take preventive measure in respect of their children while older women tend to take curative measure. With respect to education, results show that children whose mothers have primary and secondary or higher education are more likely to receive measles vaccine and seek medical care whenever they have fever or cough and diarrhoea. This indicates that education plays a significant role in mothers’ health care seeking behaviour for their children. Studies have shown that there is a direct relationship between education and mother’s health care seeking behaviour [19–22]. This may be attributed to the fact that education exposes women to the importance of seeking health care for children. There seems to be a similar relationship in respect of household wealth as children from rich households are more likely to receive measles vaccine compared to children from poor households [23, 24]. However, children from richest households are less likely to seek medical care when they have diarrhoea. This may be linked to the fact that children from richest households may be enjoying alternative medical care such as home treatment by medical personnel. Employment status of mothers has a significant impact on health care seeking behaviour for their children as working women are more likely to present their children for measles vaccine and more likely to seek medical care when their children have fever or cough [25–27]. It may be that working women have the ability to pay for treatment whenever they present their children for medical care, something which the non-working women may not be able to do on their own.

Results further show that children in urban areas are less likely to receive measles vaccine and less likely to seek medical care when down with fever or cough [28]. This indicates that children from rural areas are more likely to receive measles vaccine and seek medical care compared to their urban counterparts. Although it is expected that children in urban areas would benefit more from medical care based on the number of health facility therein, rural dwellers may have benefitted from awareness campaign by health workers on importance of consulting medical personnel for immunization and when children are sick. Child’s age is another factor that influenced health seeking behaviour of women for their children [29]. Compared to children who are 7–11 months old, children who are 12 months old and above are less likely to seek medical care when they are sick. This implies that mothers tend to pay more attention to infants than older children. This may be related to general notion that infants are yet to build immunity and as such they are more vulnerable to diseases. With respect to birth order, children of second and higher order of birth are less likely to receive measles vaccine compared to children of first order of birth [13, 30]. Whereas children of second and higher order of birth are more likely to seek medical care when they have diarrhoea. This indicates that when it comes to immunization, children of first order of birth are given preference by mothers while children of second and higher order of birth are given preference when it comes to seeking medical care during sickness.

Mothers seem to pay less attention to children who are large at birth as children who are of average size and those who are of small are more likely to receive measles vaccine and seek medical care for diarrhoea respectively. It may be that mothers perceive children who are large at birth to be strong compared to children who are either small or of average size. In fact, other studies [31] have opined that mothers considered small children to be fragile and as a result, they feel reluctant to present them for immunization. Antenatal care attendance tends to contribute positively to mothers’ health care seeking behaviour for their children. Children of women who attended antenatal care are more likely to receive measles vaccine and seek medical care when sick. Antenatal care attendance affords women the opportunity to obtain information on maternal and child health promotion.

### Policy implications

This study has shown that improvement in child health in sub-Saharan Africa requires improvement in health care seeking behaviour of women in respect of their children. Based on the findings from the study, some measures need to be taken in order to improve child health in the region. There is a need for adequate orientation and awareness creation for women at local level. Such awareness programme should be geared towards provision of useful information on issues relating to child health and enlightenment on beliefs that may be harmful to child wellbeing. For instance, young and older women should be enjoined to give priority to any aspect of their children’s health, be it immunization or seeking medical care for any ailment. At the same time, women who are of the habit of not attending antenatal care whenever they are pregnant should be encouraged to attend in order to benefit from the information provided during the clinics. Also, emphasis should be placed on seeking medical care and taking up immunization for children irrespective of their size, order of birth or age. This will go a long way in eradicating preference on the basis of such factors. Efforts should be made by governments at all levels to increase proportions of educated women in the region. Although there have been programmes in different countries on improving girl-child education, areas of improvement should be maintained while areas where there are issues should be reviewed. Governments should also look into provision of employment opportunities for women who are willing to work. For those who may wish to embark on business activities or artisanship such as tailoring, hairdressing, weaving, catering services, etc. funds should be provided for them with convenient repayment period.

### Study strengths and weaknesses

This study has made use of data from cross-sectional surveys which make the establishment of cause-effect to be difficult. The data sets for the countries were pooled together for easy analysis and interpretation. Some variables which might be of importance to the study were dropped because some countries did not measure them. Being hierarchical data sets, the use of multilevel analysis would have taken care of the structure. But this was not considered in the study. However, despite these shortcomings, the study is among the few that examined the relationship between mothers’ health care seeking behaviour and child health at sub-Saharan African level. At the same time, the study has used large data sets which include many countries and which has made the results more robust. In view of this, results obtained can be related to other countries or regions with similar characteristics.

## Conclusions

This study has revealed that mothers’ health care seeking behaviour for their children is influenced by social, maternal and child factors. Any intervention aimed at improving child health in sub-Sharan Africa should take these factors into consideration.

## Data Availability

Data used in this study were obtained from the DHS Program and available at: https://dhsprogram.com/data/available-datasets.cfm.

## Abbreviations

DHS: Demographic and Health Survey
IMCI: Integrated Management of Childhood Illness
PSU: Primary Sampling Unit
UNICEF: United Nations International Children’s Emergency Fund
WHO: World Health Organization
